# 

**Published:** 1893-02

**Authors:** 


CONTENTS

Vol. 40 No. 2.

| | PAGE. |
|---|---|
| Hygiene of Old Age | 25 |
| Cross-Thinkers | 28 |
| Are Animals Immortal? | 30 |
| Behind the Counter | 32 |
| Rural Life | 33 |
| When to Marry | 34 |
| Squinting | 36 |
| To Restore Drowning Persons | 37 |
| To Boys who Smoke | 37 |
| Dust | 38 |
| The Baby's Airing | 39 |
| Water as a Medicine | 39 |
| Beef Tea | 40 |
| Gruel | 40 |
| Mesmerism Extraordinary | 41 |
| The Age of the Earth | 42 |
| Manners for Children | 43 |
| Thomas Carlyle—A Fragment | 44 |
| Miscellaneous | 45 |
| Literary | 47 |
| It is not Much | 48 |

INDEX TO ADVERTISEMENTS OF HALF A PAGE AND OVER.

| | PAGE. |
|---|---|
| Dr. Jaeger's Sanitary Woolens | I |
| National Mutual Insurance Co. | II |
| The Press Claims Co. | III |
| The Rip Van Winkle Rocker | IV |
| James Vick's Sons | V |
| Hall's Journal Premiums. | VII |
| Washington Improvement Co. | VIII |
| J. Fehr's Compound Talcum. | IX |
| Herba Vita | X |
| Royal Baking Powder | XI |
| Buffalo Lithia Water | XI |

